# Gaze Error Estimation and Linear Transformation to Improve Accuracy of Video-Based Eye Trackers

**DOI:** 10.3390/vision9020029

**Published:** 2025-04-03

**Authors:** Varun Padikal, Alex Plonkowski, Penelope F. Lawton, Laura K. Young, Jenny C. A. Read

**Affiliations:** 1 Department of Biosciences, Newcastle University, Newcastle upon Tyne NE1 7RU, UK; penny.lawton@newcastle.ac.uk (P.F.L.); laura.k.young@newcastle.ac.uk (L.K.Y.); jenny.read@newcastle.ac.uk (J.C.A.R.); 2 School of Medicine, Newcastle University, Newcastle upon Tyne NE1 7RU, UK; alexander.plonkowski@medadvisor-operationsmile.org

**Keywords:** accuracy error, video-based eye trackers, psychophysics, EyeLink 100 Plus, Tobii Pro Nano

## Abstract

Eye tracking technology plays a crucial role in various fields such as psychology, medical training, marketing, and human–computer interaction. However, achieving high accuracy over a larger field of view in eye tracking systems remains a significant challenge, both in free viewing and in a head-stabilized condition. In this paper, we propose a simple approach to improve the accuracy of video-based eye trackers through the implementation of linear coordinate transformations. This method involves applying stretching, shearing, translation, or their combinations to correct gaze accuracy errors. Our investigation shows that re-calibrating the eye tracker via linear transformations significantly improves the accuracy of video-based tracker over a large field of view.

## 1. Introduction

Eye-tracking as a research modality continues to increase in popularity as the underlying technology continues to improve and become more accessible. It has been utilized in multiple different fields, including marketing images [1,2], human behavior and interaction [3], and medical training and research [4,5,6]. Within these applications, the precision and accuracy of the eye tracking system used are crucial factors in accurate data reporting. When evaluating subtle differences in fixation, such as those present in marketing materials or reconstructive surgery, even minor errors in recorded fixation points can lead to misleading conclusions about individuals’ focal points of attention. The phenomenon of ‘vertical drift’ or ‘calibration drift’ has been previously described within the eye-tracking literature as an increase in error of fixation registration during an experiment [7,8]. Several studied have also investigated the factors affecting the accuracy of the eye tracker [9,10]. Random offset in the gaze location can be either attributed to illumination variation between calibration and the data collection period [10] or due to variation in pupil size [11,12]. Systematic errors arise due to error during calibration or an improper mapping function used during calibration process [13,14]. Collectively, these findings highlight that eye-tracking accuracy is influenced by a combination of calibration conditions, pupil-size variations, and tracking algorithms. However, systematic errors can be corrected using the appropriate transformation methods. Furthermore, within the field of eye-tracking research, there has been work published detailing the importance of a standardized outcome reporting for increasing comparability and reproducibility. Careful consideration of these factors highlights the need for a simple methodological tool for measurement and correction of any deviation that is present immediately after calibration. This correction is assumed to remain valid, provided that no further deviations occur during the experiment. The objective of this study was to investigate the presence of any drift or deviation, as well as implement a deviation correction protocol among different eye-tracking systems. Such a protocol could be useful for other researchers in improving the accuracy of future studies, as well as potentially helping with the standardization of future work.

Most video-based eye trackers used for psychophysical experiments consist of an infrared (IR) illuminator and use the pupil-glint vector as the basis for tracking gaze location. These trackers operate with a bright or dark pupil configuration. In the bright-pupil configuration, the illuminator is close to the imaging axis, so the light is reflected off the retina, which makes the pupil appear bright (the red-eye effect). In the dark pupil mode, the IR light source is kept away from the imaging axis, which causes the pupil to appear dark with a specular reflection from the cornea known as the first Purkinje reflection. As long as the illuminator is stationary, the corneal reflection stays fixed in the image while the pupil moves in the direction of the gaze. Some trackers also incorporate a 3D model of the eye to further improve gaze location prediction accuracy [15]. During calibration, the eye tracker’s software maps the pupil-corneal vector from the camera’s coordinate space to display coordinates, processing the data before they are made available to users. Both linear and non-linear mapping have their own pros and cons [16]. Nonlinear mapping corrects curved distortion and adjusts scales smoothly across the screen; however, the error due to the squared terms in the equation increases considerably at the screen’s edge, so small variations in gaze location at the corners can result in a substantial error in gaze position.

To assess accuracy, we used two eye trackers: the EyeLink 1000 Plus (SR Research, Ottawa, ON, Canada) a state-of-the-art video-based tracker often used for psychophysics experiments, and the more affordable Tobii Pro Nano (Tobii AB, Danderyd, Sweden) eye tracker. The experiments were conducted under two different conditions. Experiment 1, performed in a field setting using the Tobii Pro Nano eye trackerwhere neither the display luminosity nor the room luminosity were calibrated and the experiment was performed without any head stabilization, was led by author AP. Experiment 2, conducted in a controlled environment using the EyeLink 1000 Plus eye tracker, was led by author V.P. Despite the variance in settings, equipment, and data filtering methods tailored to distinct research objectives, both experiments yielded a consistent outcome that the video-based eye trackers have significant and persistent biases even after calibration. We found that in both cases, these biases could be reduced by applying a linear coordinate transformation, producing a more accurate estimate of where an individual was looking on the display.

This paper presents an investigation of systematic accuracy errors in the Tobii Pro Nano and EyeLink 1000 Plus eye trackers. In our methodology, we detail the experimental setup, data collection procedures, and preprocessing techniques. The analysis section examines accuracy errors and explores different linear coordinate transformation techniques to reduce the systematic errors. In our results, we demonstrate the impact of these corrections on accuracy of the eye tracker. Finally, in the Discussion and Conclusion sections, we describe the optimal transformation technique, validate our findings, and highlight their importance and limitations.

## 2. Methods

### 2.1. Participant Demographics and Ethical Approval

For Experiment 1, there were 149 participants, of whom 78 were male and 71 were female. For Experiment 2, we had 15 healthy individuals, 7 of whom were male and 8 female, aged between 18 and 24 years, and one female in her 50s. All participants wore their habitual visual correction, while those without correction self-reported having normal visual acuity. The experimental procedures adhered to the guidelines outlined in the Declaration of Helsinki. Informed consent was obtained from each participant, and data were anonymized using unique participant IDs. Ethical approval for both experiments was granted by Newcastle University’s Faculty of Medical Sciences Ethics Committee (FMS Ethics ID: [FMS 2333] for the study led by AP and [FMS 2447/27342] for the study led by author V.P.).

### 2.2. Experiment 1

#### 2.2.1. Experimental Setup

**Figure 1:**
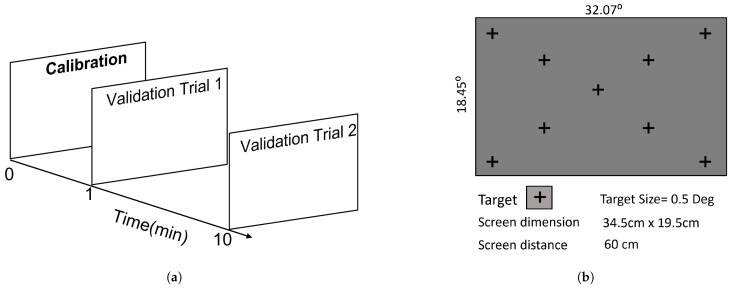
The figure shows the experimental setup for Tobii Pro Nano. The panel (**a**) shows the designed psychophysical experiment to collect gaze location. Panel (**b**) show the presented target and their location on the screen.

We collected gaze data at different locations on the LCD display with a resolution of 1920 × 1080 using the Tobii Pro Nano [15]. The participant’s head was not stabilized, and the distance to the display was approximately kept to 60 cm. The display size was 34.5 × 19.5 cm, spanning about 32 × 18 degrees of the visual field, as shown in Figure 1. The Tobii Pro Nano eye tracker was set to operate in binocular mode with a temporal sampling rate of 60 Hz.

#### 2.2.2. Procedure

The target chosen was a normal fixational cross of size with 0.5 degrees in diameter. We displayed 9 targets that were positioned diagonally, as shown in Figure 1. Only a single target was displayed at a given moment, and the code subsequently displayed the next target when it determined that the participants had fixated on the former for 1 s. Since there is no inbuilt validation process in the Tobii Pro Nano, it was not possible to determine if there were any faults during the calibration process. We recorded the gaze location binocularly. To estimate the gaze error, two validation trials per participant were taken, one at the beginning of the experiment and another at the end of 10 min, as shown in Figure 1. This procedure forms one block of data, which includes two validation trials performed after a single calibration session. During this 10-min duration, participants were engaged in viewing images of faces under a free viewing condition as part of a different psychophysics experiment. Every participant performed the experiment once; hence, we have 149 blocks of data with each of the 2 validation trials. Out of 149 data blocks, we discarded 9 blocks, either due to tracking failure or the subject not properly fixating on the target, and 140 trials were considered for further analysis.

#### 2.2.3. Data Cleaning

The gaze location of both eyes is averaged and if the disparity is greater than 5 degrees on the X axis or 1.6 degrees on the Y axis, the fixation data point is ignored. Such large disparities are physiologically improbable and are therefore likely to result from eye tracker errors. Due to the fixational eye movements and the data recording methods of eye trackers, which classify periods between microsaccades as fixations, multiple fixation locations can be registered by an eye tracker for a single target. For this study the very first fixation registered by the eye tracker, when the target is displayed, is taken as the fixation location for that given target.

### 2.3. Experiment 2

#### 2.3.1. Experimental Setup

**Figure 2:**
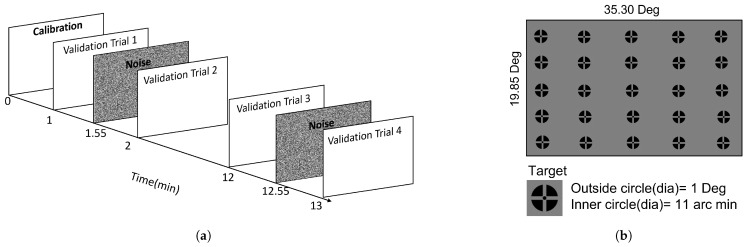
The figure shows the experimental setup for EyeLink 1000 Plus. (**a**) Experimental setup for EyeLink 1000 Plus. (**b**) Target location in EyeLink 1000 Plus.

The EyeLink 1000 Plus [17] was used in a tower-mount configuration with a chin rest to stabilize head movement, and the tracker was set to operate in binocular mode with a temporal sampling rate of 1000 Hz. The gaze data at different visual fields were acquired by presenting a target at different locations on a CRS Display (Display++) with a resolution of 1920 × 1080, which was kept 1.1 m from the participant, and the display dimensions are 70 × 35 cm, thus, the display spans 35 × 20 degrees of the total visual field. The target stimulus chosen in the experiment comprises a bull’s eye with a cross, designed to facilitate consistent fixation [18]. Unlike Experiment 1, where only 9 data points across the screen were collected per validation trial, here, the targets are presented on a 5 × 5 grid on the screen, and each target is randomized and displayed in turn, as shown in Figure 2.

#### 2.3.2. Procedure

Prior to measuring gaze error at different locations, the eye tracker was calibrated and validated using the Hv9 configuration. This configuration is a 9-point calibration and validation setup, which is one of several standard procedures available from EyeLink [19]. Only good validation (worst point error ≤ 1.5° and average error ≤ 1.0°) or fair validation (1.5° ≤ worst point error ≤ 2.0°, and 1.0° ≤ average error ≤ 1.5°) were considered acceptable and used during the experiment. In the event of poor validation, the illuminator and chin rest were adjusted to re-calibrate the tracker. The tracking was based on both pupil position and corneal reflection, and the centroid of the pupil was taken into consideration instead of fitting an ellipsoid, as it offers better precision [9]. Similar to Experiment 1, we define a block as a series of validation trials taken without the subject moving their head from the chin rest as they were performing other psychophysics tasks, as shown in Figure 2. Four validation trials (V1, V2, V3, and V4) were obtained per block: two initial consecutive trials at the beginning of the experiment and two subsequent trials after a 10-min interval. The duration of each validation trial was 50 s, since each target stimulus was displayed for two seconds. Between two consecutive trials, a 5-s duration of random noise was presented to the participant to prevent any afterimages, as shown in Figure 2. Four such blocks of trials were obtained from each participant. Given that the tracking was carried out binocularly, we considered the right and left eyes to be separate sets of data. Hence, 4 blocks from 15 participants with 2 separate sets of data from both eyes give 120 blocks. Out of the 120 blocks of trials analyzed for this study, 21 were excluded due to the tracker losing the pupil or the participant not fixating on the target; therefore, 99 useful blocks are analyzed in this study.

#### 2.3.3. Data Cleaning

Filtering the gaze location from the EyeLink 1000 Plus data was performed as follows: During the 2-s period when the participant fixated on the target, we selected the number of fixation points based on the EyeLink 1000 Plus default threshold of fixation (saccade velocity threshold ≤ 30 deg/s). Since the duration of the target on screen was 2 s, we normally had multiple fixation points for a given target, as the time periods between two microsaccades are defined as single fixation points. Filtering the points is performed in different steps. The first stage is to determine whether the duration of any fixation point exceeds 0.8 s; if so, we have considered the location of this point to be the fixation location for the given target; if no such fixation points are present, then we check for duration above 0.5 s and repeat the process. If several fixation points exceeded the given threshold duration, or if there were no fixation points above 0.5 s, we selected the fixation location that was closest to the displayed target.

## 3. Accuracy Error

**Figure 3:**
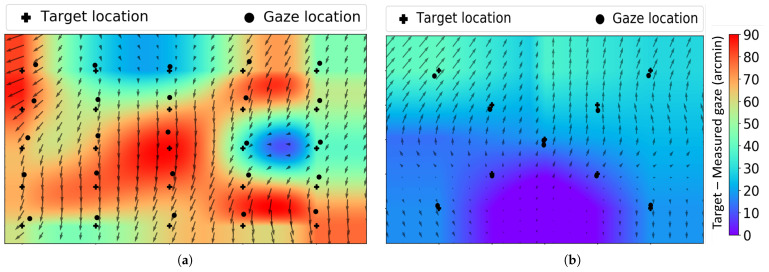
Accuracy errors in video-based eye trackers. (**a**) shows the accuracy error in EyeLink 1000 Plus. (**b**) shows the accuracy error in Tobii Pro Nano. The black cross and black dots are the target location and fixation gaze location, respectively. The color map indicates the interpolated offset values, and the arrows indicate the correction required to minimize the error.

Once the fixation point was obtained for all the displayed targets, we calculated the shift between the displayed target position and the corresponding fixation location, which indicates the accuracy error in the tracker. Figure 3 depicts the accuracy error of a singular trial from both the EyeLink 1000 Plus and Tobii Pro Nano. The black cross represents fixation target locations, and the black dots represent the fixation gaze locations reported by the eye tracker. The regions between these dots are 2D cubic interpolated, and this is displayed as a continuous color map. It is clear that both the Tobii Pro Nano and EyeLink 1000 Plus do possess systematic errors. For instance, considering the EyeLink 1000 Plus accuracy error in Figure 3, the majority of the fixation gaze data are positioned above the target location, indicating a systematic offset in the upward direction of the displayed target. Similar results are seen from the Tobii Pro Nano eye tracker, where the gaze locations at most of the points are registered below the target locations, showing systematic offset in a downward direction, as shown in Figure 3. These systematic offsets cannot be attributed to individual participant peculiarities, such as consistently looking slightly below fixation targets. The calibration process assume that participants look directly at the targets, regardless of their position on the display. Therefore, any individual biases should have been corrected during the inbuilt calibration process. In these trials, the major part of the error can be corrected by vertically translating the frame downward for the EyeLink 1000 Plus trial and vertically translating the frame upward in the Tobii Pro Nano trial, as indicated by the arrows in Figure 3. Most of the offsets in the gaze location exhibited a similar consistent pattern. This observation indicated that the errors are systematic and probably a result of inaccurate calibration of the video-based tracker, and it is worth correcting these errors in post processing using the coordinate transformation technique described in Section 4.

In the analysis of 140 trials from the Tobii Pro Nano tracker, the average RMS error values of the offsets were 32.60 arcmin for validation trial 1 and 41.14 arcmin for validation trial 2. For the EyeLink 1000 Plus tracker, where 99 trials were analyzed, the mean RMS values of the offsets were 45.01 arcmin, 48.08 arcmin, 48.09 arcmin, and 51.68 arcmin for validation trials 1, 2, 3, and 4, respectively. Thus, the average RMS systematic error was greater than 0.5 degrees in both tracker.

## 4. Coordinate Transformation

As seen in Section 3, video-based trackers generally have an offset in the gaze location from the corresponding fixation target. From Figure 3, it is clear that these offsets can be corrected by linearly translating the coordinate space. We performed a linear transformation in which we improved these offsets between gaze location and target location by stretching, shearing, rotating, or translating the coordinate space.

The similar approach of using linear transformation and linear regression techniques to improve the accuracy of the eye tracker is discussed in [20,21]. We have analyzed different coordinate transformations to select the one that better minimizes the offset between the gaze and target location. The coordinate transformation techniques explored in this study are as follows:

- Stretch–Shear
- Stretch–Translate
- Affine (stretch, shear, and translate)

In the case of the stretch and shear transformation, the optimization is performed with a 2 × 2 matrix, as shown in Equation (1).

(1) $$\left[\begin{array}{c} x_{new}\\ y_{new} \end{array}\right]=\left[\begin{array}{cc} a & b\\ c & d \end{array}\right]\left[\begin{array}{c} x_{pos}\\ y_{pos} \end{array}\right]$$

The terms *a* and *d* stretch and the terms *c* and *b* shear the coordinate space. It is also important to note that these matrices also contribute to rotating the coordinate space. The Nelder–Mead simplex algorithm is used to optimize a,b,c, and d terms in matrix (2) so that the RMS difference between the target location and gaze location is minimal for a given validation trial. The affine transformation involves translation in addition to stretching and shearing of the space, as shown in Equation (2).

(2) $$\left[\begin{array}{c} x_{new}\\ y_{new} \end{array}\right]=\left[\begin{array}{ccc} a & b & T_{x}\\ c & d & T_{y} \end{array}\right]\left[\begin{array}{c} x_{pos}\\ y_{pos}\\ 1 \end{array}\right]$$

Here, similar to stretch and shear transformation, the terms *a*, *b*, *c*, and *d* are stretching, shearing, and rotating, whereas the terms $T_{x}$ and $T_{y}$ are the translation in the *x* and *y* positions, respectively. Further setting the initial condition during optimization is essential, as six parameters are being optimized, unlike four in the stretch–shear transformation. The initial condition is set for $T_{x}$ and $T_{y}$ as the mean difference between the *x* and *y* coordinates and their corresponding gaze locations, respectively. Another subset of an affine transformation that we analyzed is stretch and translate. The shear terms, which are *b* and *c* in Equation (2), are set to zero. Hence, we have four parameters to optimize, which are *a*, *d*, $T_{x}$, and $T_{y}$.

## 5. Results

Our primary question was whether the differences between recorded gaze positions and intended targets reflect random variability or consistent biases. To investigate this, we recorded gaze positions at multiple time points (validation trials 1 and 2 in Experiment 1 and validation trials 1, 2, 3, and 4 in Experiment 2). It was important to determine whether a transformation derived from validation trial 1 would reduce the error in subsequent validation trials. While the transformation was guarantee to improve or at least maintain accuracy in validation trial 1, as it was specifically tuned for that trial, its effectiveness in later trials would indicate the presence of consistent biases rather than random noise. If the errors were purely random, a transformation optimized for validation trial 1 would not be expected to reduce the error in subsequent trials. To test this, we identified the optimal transformation for validation trial 1 and applied it to the other validation trials.

### 5.1. Experiment 1: Coordinate Transformation Results

**Figure 4:**
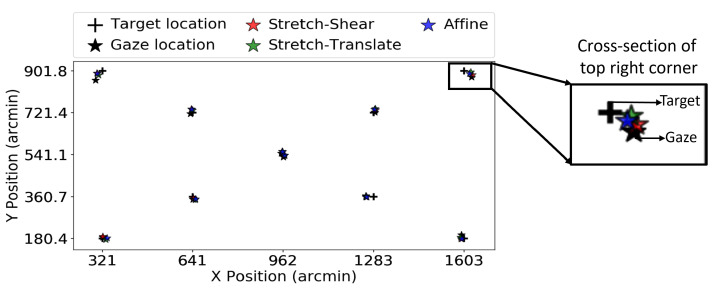
The figure shows data from the Tobii Pro Nano eye tracker for validation trial 1, where the black cross represents the target location and the black dots indicate the raw gaze locations. The colored stars are the target locations after applying the corresponding coordinate transformation. The transformed gaze (colored stars) is closer to the target (cross) than the untransformed gaze (black star), which is expected here since the transforms were fitted to these data.

**Figure 5:**
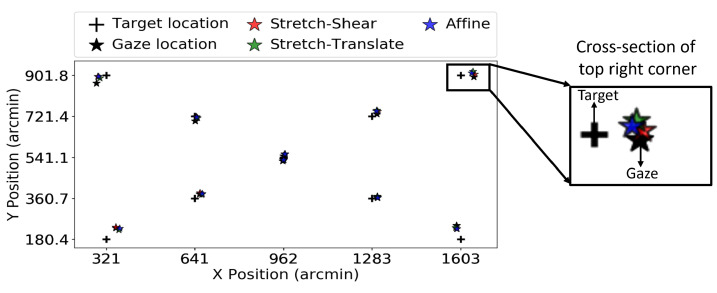
The figure shows data from the Tobii Pro Nano eye tracker for validation trial 2, where the black cross represents the target location and the black dots indicate the raw gaze locations. The colored stars are the target locations after applying the corresponding coordinate transformation. Similar to Figure 4, the transformed gaze locations are systematically closer to the target, despite the used transformation being optimized to an independent dataset collected over 10 min prior.

The target location, fixation gaze location, and transformed gaze location of a randomly selected participant from the Tobii eye tracker are shown in Figure 4. Here, the target locations are marked with crosses, while the fixation gaze locations are represented by black stars. After applying the stretch–shear, stretch–translate, and affine transformations to the fixation gaze locations, the newly transformed locations are plotted as stars in red, green, and blue, respectively. The impact of these transformations on validation trial 2 is shown in Figure 5. It is important to note that the transformations continue to have the effect of bringing the measured gaze closer to the target, despite the fact that they were fitted to a distinct set of data. To determine whether the consistent improvements observed in Figure 4 and Figure 5 are representative, we analyzed the RMS error across all 140 data blocks recorded by the Tobii eye tracker.

**Figure 6:**
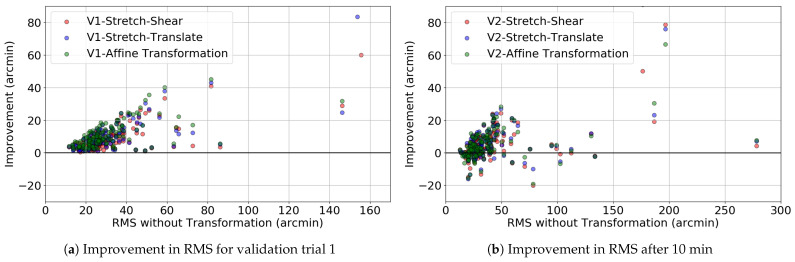
Improvement in RMS after applying linear coordinate transformations. (**a**) Validation trial 1, conducted at the beginning of the experiment. Each point shows the average improvement across all 9 target locations, with all results above zero, improving or maintaining error. (**b**) Validation trial after 10 min, showing that systematic calibration offsets persist for at least 10 min, with most fixations showing positive improvements.

Figure 6a shows the improvement in the RMS error for validation trial 1, i.e., the data to which the transformation was fitted. As expected, all improvements are positive. The mean improvement is greatest for the 6-degree-of-freedom (6-DoF) affine transformation (12.6 arcmin), followed by the 4-DoF stretch–translate transformation (10.4 arcmin), and is smallest for the 4-DoF stretch–shear transformation (8.9 arcmin). To determine whether same transformation matrices still improve the RMS value after 10 min of time, we applied the transformation matrix obtained from validation trial 1 to gaze location in validation trial 2, then plotted the RMS, which is shown in Figure 6b. Even though a few points on the y-axis are negative, indicating increased error due to the transformation, the majority of transformed points have positive values. The mean improvements in RMS error were 4.47, 5.70, and 5.66 arcmin for the stretch–shear, stretch–translate, and affine transformations, respectively, while the median improvements were 2.56, 3.76, and 3.39 arcmin. The maximum individual improvements reached 78 arcmin for the stretch–shear transformation, 90 arcmin for the stretch–translate transformation, and 92 arcmin for the affine transformation. Thus, the errors in recorded eye position were on average reduced, indicating that the transformation fitted on validation trial 1 captured persistent biases.

Furthermore, we performed a Wilcoxon signed-rank test to determine if the improvement in the validation trial 2 seen after transformation is a significant different from the median value 0. We performed a non-parametric test as the distribution was not normally distributed. We found that the improvement after stretch–shear, stretch–translate, and affine transformation are all significantly different from the median value (*p*-value < 0.01). This indicates that the transformation matrix obtained from the V1 validation trial significantly improves the RMS error. This further supports our claim of the presence of systematic error and validates our technique of using linear coordinate transformation to reduce these errors.

### 5.2. Experiment 2: Coordinate Transformation Results

The Tobii Pro is a relatively low-cost eye tracker, retailing for approximately £1000. It is therefore not surprising that its calibration process may introduce systematic biases in gaze location measurements. We ran a more detailed version of the experiment on the EyeLink 1000 Plus eye tracker, a research-grade eye tracker that costs around £20,000. The EyeLink 1000 Plus acknowledges the possibility of drift in gaze location over time, which it corrects by translating the calibration frame using newly acquired gaze data presented at the center of the screen. To assess the effectiveness of this drift correction, we manually performed the procedure and compared its impact against the linear coordinate transformation techniques explored in the paper.

**Figure 7:**
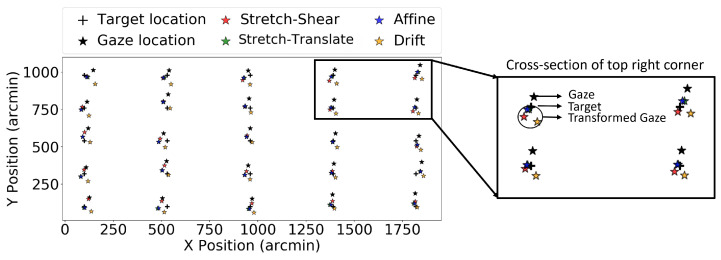
The figure shows data from the EyeLink 1000 Plus eye tracker for validation trial 1, where the black cross represents the target location and the black dots indicate the raw gaze locations. The colored stars are the target locations after applying the corresponding coordinate transformation. The transformed gaze (colored stars) is systematically closer to the target (cross) than the untransformed gaze (black star), which is expected since the transforms were fitted to these data.

**Figure 8:**
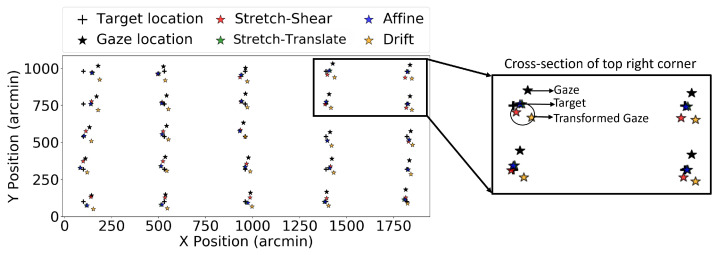
The figure shows data from the EyeLink 1000 Plus eye tracker for validation trial 4, where the black cross represents the target location and the black dots indicate the raw gaze locations. The colored stars are the target locations after applying the corresponding coordinate transformation. Similar to Figure 7, the transformed gaze locations are systematically closer to the target, although the used transformation is optimized to an independent dataset collected over 10 min prior.

**Figure 9:**
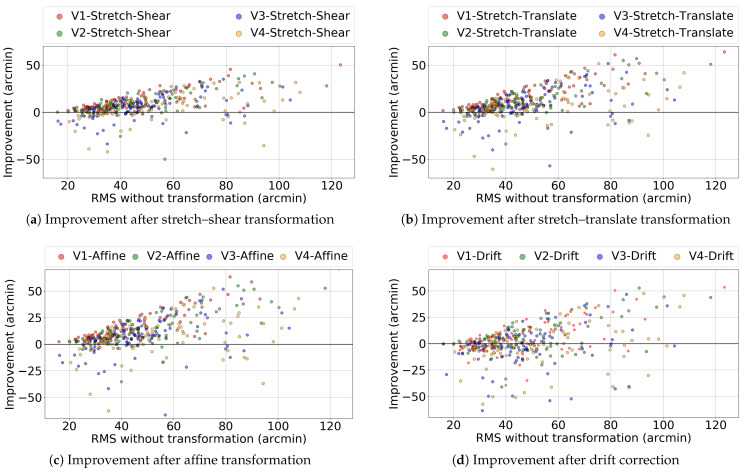
Comparison of improvement after different transformations. Validation trials V1 and V2 were taken at the beginning of the experiment, while trials V3 and V4 were taken after 10 min. (**a**) shows improvement after stretch–translate transformation. (**b**) shows improvement after stretch–translate transformation. (**c**) shows improvement after affine transformation and (**d**) shows the drift correction performed on the gaze location.

Here we performed the coordinate transformation on all 98 trials obtained from 15 participants using the EyeLink 1000 Plus eye tracker to check whether these transformations performed better in the entire data set. Figure 7 shows the target location, fixation gaze location, and gaze location after different transformations for one selected participant from the EyeLink 1000 Plus eye tracker. Figure 8 shows the effect of these same transformations on validation trial 4. Note that the transformations still tend to bring the measured gaze closer to the target, even though they were fitted to a different set of data. Similarly to that in Experiment 1, we plotted the improvement in the RMS value of the gaze location before and after transformation. We independently plotted each of the three transformations in order to determine which one performed the best. Figure 9a–c show the RMS value improvement vs. RMS without transformation in stretch–shear, stretch–translate, and affine transformation, respectively. Similar to the result in Experiment 1, despite using the same transformation matrix obtained for validation trial 1 to correct validation trials 2, 3, and 4, we found that the majority of the points lie above the y = 0 line, indicating a positive improvement and thus persistent biases in reported gaze. We have also tested the reliability of the drift correction technique in reducing the RMS value, which is shown in Figure 9d. In Figure 9d, it is evident that translating the entire frame based on the shift of the center data is not efficient in reducing the RMS value, as some of the “improvements” are negative even in V1. This is due to the fact that subtracting the offset at the screen’s center does not necessarily reduce errors for all gaze points, and in some cases, it has actually increased the errors. The average improvements in RMS error for validation trial 2 were 4.44, 5.76, and 6.00 arcmin for the stretch–shear, stretch–translate, and affine transformations, respectively, while the median improvements were 3.87, 5.04, and 5.20 arcmin. The max individual improvements reached 31 arcmin for the stretch–shear transformation, 51 arcmin for the stretch–translate transformation, and 52 arcmin for the affine transformation. Even in validation trial 4, the improvement remained significant (Wilcoxon signed-rank test, *p* < 0.001 for stretch–shear, stretch–translate and affine transformations fitted to the validation trial).

**Figure 10:**
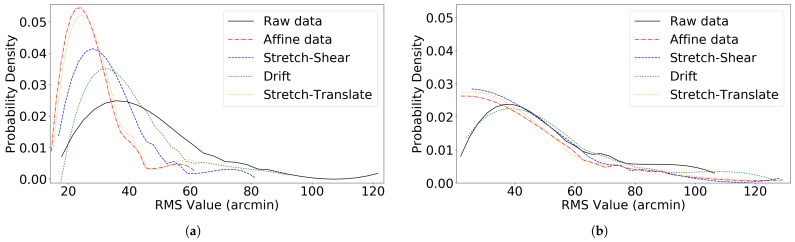
Distribution of RMS values before and after linear coordinate transformations in the EyeLink 1000 Plus eye tracker. (**a**) Validation trial 1. (**b**) Validation trial 4, performed on 98 data blocks.

**Figure 11:**
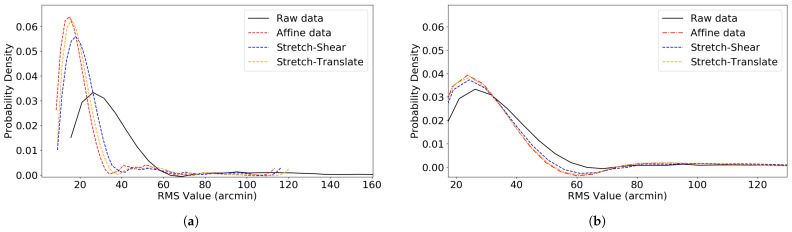
Distribution of RMS values before and after linear coordinate transformations in the Tobii Pro Nano eye tracker. (**a**) RMS distribution for validation trial 1. (**b**) RMS distribution for validation trial 2, performed on 140 data blocks.

The Figure 10 and Figure 11 show the RMS error distribution before and after transformation in both EyeLink 1000 Plus and Tobii Pro Nano eye trackers. The RMS value and the max value for all validation trials for both EyeLink 1000 Plus and Tobii Pro Nano are given in Table 1.

## 6. Discussion

As previously stated in Section 3, it is evident that accuracy errors greater than 0.5 degrees persist in both video-based trackers. With a 12pt width font size (where each word is about 7.5 to 10 mm wide) and with a viewing distance of 43 to 57 cm, a 0.5 degree gaze error could shift the gaze location by nearly half a word’s width. This error might cause the tracker to wrongly register a gaze on the neighboring word rather than the actual one.

The mean and maximum values of the RMS distribution before and after correction are given in Table 1. It is clear from the table that the errors before correction are slightly worse on the Eyelink 1000 Plus than on the Tobii Pro Nano. From a further look at the results of the affine transformation, an average RMS improvement of 12.04 and 5.66 arcmin is seen for validation trials 1 and 2 in the Tobii Pro Nano eye tracker, and improvements of 17.21 and 6.0 arcmin are seen for validation trials 1 and 4 in the EyeLink 1000 Plus tracker.

It is important to understand if these offsets can be corrected in post-processing. This issue has been addressed in various studies using different transformations. For instance, stretch and shear transformations have been used to correct these errors [20]. In our study, we found that among the analyzed transformations, the affine transformation performed the best. This is expected, as the affine transformation has six free variables that allow for stretching, shearing, rotating, and translating the coordinate space. Despite having only four parameters to optimize in both the stretch–shear and stretch–translate transformations, being a subset of the more comprehensive affine transformation, it is also clear from Table 1 that stretching and translating the space reduces the overall RMS error more effectively than stretching and shearing the coordinate space. This also suggest that translating the space is better over shearing alongside with stretching unlike the stretching and shearing approach. Another approach given in the literature is to measure the offset between the target and the recorded gaze location and apply a region-dependent error correction to improve fixation accuracy without relying on a global linear transformation model [22].

The RMS errors are generally smaller after the transformation, with notably fewer large errors at V1. Even though at V4 there are some large errors, in general, there are many more small errors than before the transformation. As expected, the 6-parameter affine transformation reduces errors the most. However, the strong performance of stretch–translate suggests that these parameters are doing most of the work. Conversely, stretch–shear is less effective. Furthermore, drift correction shows essentially no benefit after the first trial.

From the 99 blocks of data analyzed using the EyeLink 1000 Plus, we found that the transformation obtained at the beginning of the experiment (for validation trial 1) had positive improvement in 70.1% of the cases. Similarly, in the case of the Tobii tracker, we analyzed 140 blocks of data, out of which 73.3% showed positive improvement on validation 2 with the initial transformation. These high percentages demonstrate the initial transformation’s effectiveness across a significant portion of our data. However, it is crucial to note that systematic errors in the tracker are not static over extended periods. Our analysis, as indicated in Figure 10 and Figure 11, shows that these systematic errors remain consistent for less than 10 min. This implies that to effectively correct these errors using linear transformations, the transformation procedure needs to be repeated approximately every 10 min. In addition, we discovered that the overall RMS value is not improved by translating the entire frame using data from a single data point in the center alone, which we termed as drift correction here in this paper. We found it is preferable to collect multiple data points at different locations on the screen and perform a linear transformation. A similar approach of translating the entire frame by averaging errors across the screen, rather than relying on a single measurement, was implemented by Zhang et al. They divided the display into three regions (top-left, top-right, and bottom) and applied separate corrections to each. However, this method did not significantly improve accuracy compared to the average single-point correction [23].

It is also important to note that the RMS accuracy error is no better for the state of the art Eyelink 1000 Plus than for the far more affordable Tobii Pro Nano. This finding indicates that the Tobii Pro Nano provides a level of accuracy comparable to that of the EyeLink 1000 Plus, consistent with the values quoted in their technical reports: 0.25–0.5 degrees average for EyeLink and 0.3 degrees for Tobii Pro Nano. Additionally, the linear transformation method used in this study was found to be effective for both eye trackers. This suggests that the Tobii Pro Nano is a cost-effective alternative without compromising accuracy but with a trade-off in temporal resolution.

## 7. Conclusions

These systematic biases primarily manifest as a combination of stretch and translation. Correcting for these biases through a validation trial improves gaze accuracy beyond the raw calibration provided by the manufacturer, though substantial inaccuracies remain. For instance, in the EyeLink 1000 Plus, applying a transformation to the reported eye measurements reduced errors from 0.75° to no better than 0.5°. The biases persist over a timescale where a fitted transformation remains effective for approximately ten minutes. Our analysis indicates that a portion of this error can be further minimized using linear coordinate transformations. Among the transformations tested, the affine transformation significantly improved gaze accuracy for both the EyeLink 1000 Plus and Tobii Pro Nano video-based eye trackers. Notably, the stretch–translate transformation, which involves only four parameters and is therefore simpler to optimize, yielded results comparable to the affine transformation.

Although linear transformation can improve accuracy errors, its effect becomes minimal after 15 min of the initial correction. Further studies on the temporal changes in accuracy error should be conducted to accounted for the temporal variations.

## Figures and Tables

**Table 1:** RMS and Max value in arcmin before and after transformation for Tobii Pro Nano and EyeLink 1000 Plus eye tracker.

	Tobii Pro Nano Tracker RMS				EyeLink 1000 Plus RMS							
	V1 (Arcmin)		V2 (Arcmin)		V1 (Arcmin)		V2 (Arcmin)		V3 (Arcmin)		V4 (Arcmin)	
	Mean	Max	Mean	Max	Mean	Max	Mean	Max	Mean	Max	Mean	Max
Without transformation	32.60	155.56	41.14	278.29	45.01	123.28	48.08	118.05	48.09	104.24	51.68	107.88
Stretch–Shear	23.74	117.30	36.67	274.06	33.13	82.35	38.25	89.95	44.06	106.59	47.24	129.71
Stretch–Translate	22.22	121.56	35.44	271.26	29.04	62.90	35.18	73.71	42.50	113.72	45.91	118.69
Affine	20.56	114.45	35.48	270.69	27.80	62.21	34.64	75.21	42.14	123.21	45.68	131.02

## Data Availability

The data presented in this paper the accuracy of EyeLink 1000 Plus and Tobii Pro Nano can be found at here: https://doi.org/10.25405/data.ncl.28669472 . The Matlab code of the explored transformation can be found here: https://doi.org/10.25405/data.ncl.28669673.
